# Tumor Mutational Burden and Efficacy of Immune Checkpoint Inhibitors: A Systematic Review and Meta-Analysis

**DOI:** 10.3390/cancers11111798

**Published:** 2019-11-15

**Authors:** Jong Yeob Kim, Andreas Kronbichler, Michael Eisenhut, Sung Hwi Hong, Hans J. van der Vliet, Jeonghyun Kang, Jae Il Shin, Gabriele Gamerith

**Affiliations:** 1 Yonsei University College of Medicine, Seoul 03722, Korea; crossing96@yonsei.ac.kr; 2 Department of Internal Medicine IV, Medical University Innsbruck, 6020 Innsbruck, Austria; andreas.kronbichler@i-med.ac.at; 3 Luton & Dunstable University Hospital NHS Foundation Trust, Luton LU4 0DZ, UK; michael_eisenhut@yahoo.com; 4 Department of Global Health and Population, Harvard T. H. Chan School of Public Health, Boston, MA 02115, USA; sunghwihong@gmail.com; 5 Department of Medical Oncology, Amsterdam UMC, Cancer Center Amsterdam, VU University, 1081 HV Amsterdam, The Netherlands; jj.vandervliet@amsterdamumc.nl; 6 Department of Surgery, Gangnam Severance Hospital, Yonsei University College of Medicine, Seoul 06273, Korea; 7 Department of Pediatrics, Yonsei University College of Medicine, Seoul 03722, Korea; 8 Internal Medicine V, Department of Hematology & Oncology, Medical University Innsbruck, 6020 Innsbruck, Austria; gabriele.gamerith@i-med.ac.at; 9 Tyrolean Cancer Research Institute, 6020 Innsbruck, Austria

**Keywords:** tumor mutational burden, immune checkpoint inhibitors, hazard ratio, overall survival, progression-free survival, PD-1 inhibitor, PD-L1 inhibitor, CTLA-4 inhibitor

## Abstract

Tumor mutational burden (TMB) is a genomic biomarker that predicts favorable responses to immune checkpoint inhibitors (ICIs). Here, we set out to assess the predictive value of TMB on long-term survival outcomes in patients undergoing ICIs. We systematically searched PubMed, Embase, CENTRAL and clinicaltrials.gov from inception to 6 August 2019. We included retrospective studies or clinical trials of ICIs that reported hazard ratios (HRs) for overall survival (OS) and/or progression-free survival (PFS) according to TMB. Data on 5712 patients from 26 studies were included. Among patients who received ICIs, high TMB groups showed better OS (HR 0.53, 95% CI 0.42 to 0.67) and PFS (HR 0.52, 95% CI 0.40 to 0.67) compared to low TMB groups. In patients with high TMB, those who received ICIs had a better OS (HR 0.69, 95% CI 0.50 to 0.95) and PFS (HR = 0.66, 95% CI = 0.47 to 0.92) compared to those who received chemotherapy alone, while in patients with low TMB, such ICI benefits of OS or PFS were not statistically significant. In conclusion, TMB may be an effective biomarker to predict survival in patients undergoing ICI treatment. The role of TMB in identifying patient groups who may benefit from ICIs should be determined in future randomized controlled trials.

## 1. Introduction

Cancer immunotherapy using immune checkpoint inhibitors (ICIs) is approved for several malignancies [1,2]. Although some patients experience significant clinical benefit from ICIs, others have no or limited clinical benefit [3,4,5], in some cases also accompanied by severe side effects. Therefore, discovering biomarkers that can identify patients who may benefit from ICIs is crucial. The programmed death-ligand 1 (PD-L1) expression density is widely used and thus far the only biomarker in clinical routine in various cancer entities. It was approved by the US Food and Drug Administration as a companion diagnostic tool [6,7]. A recent meta-analysis demonstrated that programmed cell death protein 1 (PD-1) or PD-L1 blockade therapy could decrease the risk of death by 28% in comparison to conventional treatment. Moreover, a greater risk reduction was achieved in patients positive for PD-L1 (34%) compared to those with negative staining (20%) [8], providing robust evidence for the clinical efficacy of ICIs, especially in patients with PD-L1-positive tumors. However, PD-L1 expression as a predictive biomarker has many limitations. It may change over time or upon the initiation of treatments such as chemotherapy and radiotherapy [9]. Tumor may be show heterogenous PD-L1 expression, and small biopsies or tumor microarrays may miss PD-L1 expression and give discrepant results from surgically resected tissue samples [9]. Pre-analytical factors such as types of fixative and duration of fixation, differences in detecting techniques such as antibody reagents and immunohistochemistry platforms, differences in definitions of cut-offs and poor observer reproducibility account for different results of PD-L1 expression [9,10,11]. Positive PD-L1 may also be induced by inflammatory procedures, such as release of interferon and T-cell recognition, which can be driven by PD-1 inhibition [12]. In several phase III randomized controlled trials (RCTs), PD-L1 immunohistochemistry has not fulfilled its promise as a predictive biomarker [4,13,14,15], showing significant risk reduction rates with ICIs even in patients with negative PD-L1 staining. Therefore, it is difficult to select patients for ICI therapy using PD-L1 expression alone [8,16]. Identification of putative biomarkers that can better predict ICI efficacy and thereby allow better patient selection is of the utmost clinical need in terms of economic considerations and immune-related adverse effects [17,18,19].

Recently, tumor-specific features derived from genome-wide analysis emerged as effective biomarkers to predict the response to ICI treatment [20,21,22]. It was suggested that a higher frequency of gene mutations, denoted as tumor mutational burden (TMB), increase the likelihood of generating immunogenic tumor neoantigens recognized by the host immune system [20,21,22]. As a direct indicator of immune recognition, neoantigen load was initially studied as a promising candidate [21]. Although mutational load, neoantigen load and expression of cytolytic markers in the immune microenvironment were associated with clinical benefit after ipilimumab treatment, there was no recurrent neoantigen peptide sequence which predicted response [21]. It was recently reported that TMB is more predictive concerning the clinical benefit from ICIs than neoantigen load using either a moderate or strong threshold to patient-specific class I human leukocyte antigen (HLA) alleles [23]. Thus, TMB was investigated as an emerging biomarker for prediction of response to ICI treatments. In patients who received anti-PD-1 or anti-PD-L1 monotherapy involving 27 tumor types or subtypes, Yarchoan et al. [24] showed that the response rate correlated with TMB. Although there has been a growing body of evidence revealing a better response in patients with higher TMB treated by ICIs, studies exploring the interaction between TMB and the long-term efficacy of ICI treatments are limited by the relatively inadequate power of primary studies, mainly due to their exploratory nature.

Current investigations are attempting to validate the long-term oncologic impact of TMB from two perspectives. One is the role of TMB as a predictive biomarker of ICI treatment and the other is the efficacy of ICI treatment compared to conventional treatment in groups of patients with different TMB levels. To synthesize the currently available evidence on these subjects, we performed a systematic review and meta-analysis.

## 2. Results

### 2.1. Identification of Studies and Study Characteristics

**Figure 1:**
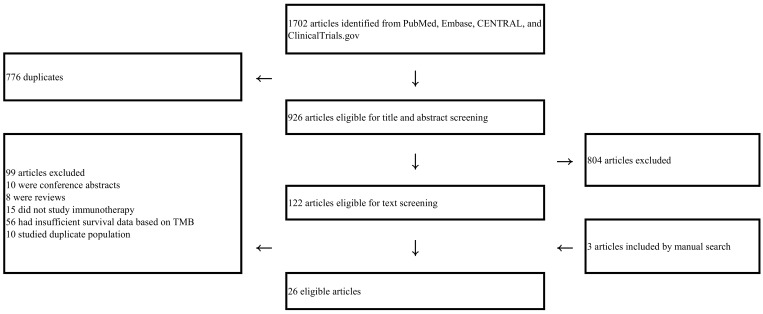
Flow of the literature search.

Our initial search retrieved 1702 publications of which 122 articles were eligible for full-text screening (Figure 1). After full-text review of 122 publications and manual search of articles, 26 studies were finally eligible, with years of publication ranging from 2014 to 2019 (Table 1 and Table 2) [5,20,21,22,23,25,26,27,28,29,30,31,32,33,34,35,36,37,38,39,40,41,42,43,44,45]. The number of patients in each study ranged from 15 to 1662. Five articles were clinical trials with prospective assessment of TMB, four of which were RCTs. The other 21 studies were retrospective studies of cohorts or clinical trials, corresponding to 27 comparison pairs (high TMB arm versus low TMB arm). Ten comparisons studied patients with melanoma, and eight comparisons studied patients with non-small cell lung cancer (NSCLC). Gastric cancer, head and neck squamous cell cancer, small-cell lung cancer and urothelial carcinoma were addressed in one comparison each. All patients included in the enrolled studies were diagnosed as having advanced or metastatic diseases. For TMB detection, 12 studies used whole-exome sequencing (WES) while 14 used next-generation sequencing (NGS). The definition of high and low TMB was heterogeneous among the studies.

### 2.2. High TMB Group Versus Low TMB Group

**Figure 2:**
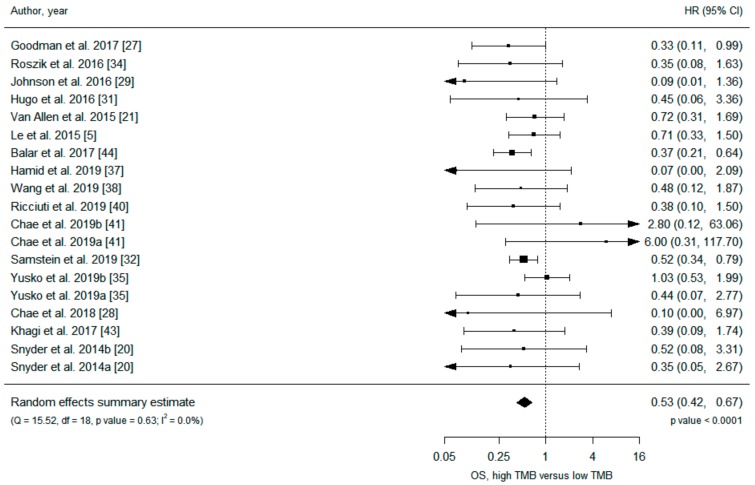
Meta-analysis of immune checkpoint inhibitor therapy and overall survival, high TMB group versus low TMB group.

**Figure 3:**
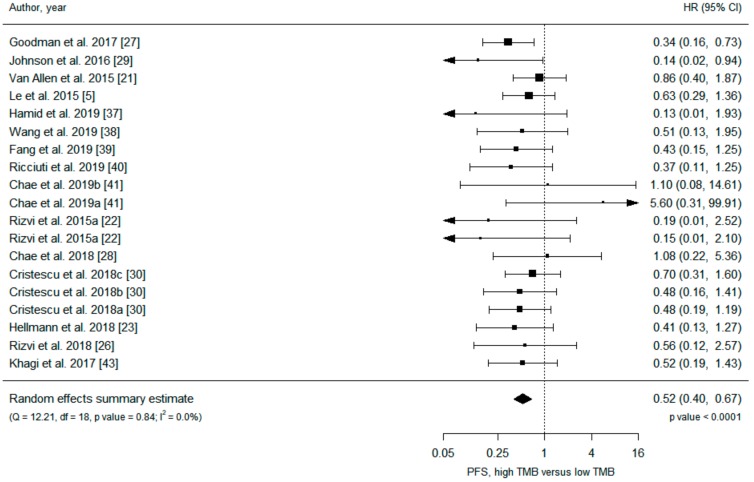
Meta-analysis of immune checkpoint inhibitor therapy and progression-free survival, high TMB group versus low TMB group.

Data on 3155 patients from 21 primary studies were included in the analysis (Table 1), and all but one [5] study were retrospective. Under a random-effects model, patients in the high TMB group receiving ICIs had significantly increased overall survival (OS) (hazard ratio (HR) 0.53, 95% confidence interval (CI) 0.42 to 0.67) and progression-free survival (PFS) (HR 0.52, 95% CI 0.40 to 0.67) compared with patients in the low TMB group (Figure 2 and Figure 3). Analysis under fixed effects showed a similar result (Table S1), and heterogeneity was low in both meta-analyses (I^2^ = 0%). There was no evidence of publication bias, with no asymmetry in the funnel plots (Figures S1 and S2).

Subgroup analyses (Table 3) revealed that PD-L1 inhibitors (HR 0.35, 0.21 to 0.61) were associated with greater OS benefit in the high TMB population when compared with PD-1 inhibitors (HR 0.62, 95% CI 0.33 to 1.17) (I^2^ among subgroups = 44%). Detection of TMB by NGS (HR 0.44, 95% CI 0.33 to 0.59) was associated with a greater OS benefit in the high TMB population when compared with detection of TMB by WES (HR 0.73, 95% CI 0.50 to 1.06) (I^2^ among subgroups = 77%), while no such significant difference was found in the PFS outcome. Association of TMB level and OS or PFS was not heterogeneous among subgroups of cancer type (melanoma versus NSCLC), sample source (tumor tissue versus blood), detection method (WES vs NGS), study design (clinical trials vs cohorts) and the number of participants. There was a trend for better OS (HR 0.66, 95% CI 0.43 to 1.01) of patients with melanoma in the high TMB group compared to the low TMB group, and higher TMB was significantly associated with better PFS in patients with NSCLC (HR 0.53, 95% CI 0.30 to 0.93). The benefit of PFS or OS was not found in subgroups of TMB detection by blood sample, but this may be due to the small sample size and subsequent imprecision of the study estimates.

### 2.3. ICI Arm Versus Chemotherapy Arm, within High TMB Group or Low TMB Group

Data on 2557 patients were included in the analysis. The patient data were based on four RCTs (Table 2), of which two RCTs prospectively analyzed TMB to test its role as a predictive biomarker. In patients with high TMB, the ICI arm showed prolonged OS (HR 0.69, 95% CI 0.50 to 0.95) and PFS (HR 0.66, 95% CI 0.47 to 0.92) compared to chemotherapy under a random-effects model (Table S1, Figures S3 and S4). In patients with low TMB, there was a tendency for prolonged OS in the ICI arm (HR 0.78, 95% CI 0.60 to 1.00), but the association was not statistically significant (*p*-value = 0.051), and there was no PFS benefit (HR 1.14, 95% CI 0.83 to 1.57) under a random-effects model (Table S1, Figures S5 and S6). Heterogeneity was low in all associations and a fixed effects model showed similar results to the random-effects model (Table S1). There was no publication bias (Figures S7–S10). A subgroup analysis was not available due to the small number of studies.

## 3. Discussion

This meta-analysis focuses on the association between TMB and long-term outcomes assessed by OS and PFS in cancer patients treated with ICIs. Integrating more than 5000 patient data with various advanced cancer types, our pooled analysis revealed a 47% risk reduction for death and a 48% risk reduction for disease progression in patients with high compared to low TMB undergoing ICI treatment. Such survival differences according to TMB level were not found in patients undergoing therapy other than ICIs [46]. The ICIs compared to chemotherapies especially resulted in prolonged OS and PFS in patients with high TMB, whereas in patients with low TMB no PFS benefit was suggested. Therefore, our study suggests that TMB may be an effective predictive biomarker for ICI therapy. Even though our data are promising, more clinical investigations are needed to decide the optimal cut-off value that should be used for adding TMB to the routine clinical practice as a predictor of clinical efficacy.

TMB, which is defined as the total number of somatic mutations in the tumor exome [24], was initially measured using WES [20,21,22]. Thus, the utility of TMB was calculated primarily for the investigational study-based cohort. Subsequently, many of the studies included in this meta-analysis were small-sized, exploratory cohorts and contained patients with a variety of malignancies. Nevertheless, the favorable influence of high TMB on the long-term survival of patients under ICIs treatment did not significantly differ among studies with large versus small sample sizes (>100 versus <100). This tendency was also maintained when studies of patients with melanoma or NSCLC were analyzed separately.

Measuring TMB by WES has some limitations in daily clinical practice due to the tissue processing difficulty, time- and labor-intensiveness due to its large sequencing capacity and subsequent high costs. Due to these difficulties, a validated hybrid capture-based NGS platform was developed and is being used with several pragmatic advantages [47,48]. However, the procedures differ in estimating the total number of somatic mutations. In WES, the germline variants are excluded after comparing data derived from normal tissue. The Foundation Medicine NGS approach, one of the most frequently used platforms, measures the number of base substitutions (synonymous and nonsynonymous mutations) in the coding region of targeted genes and defines TMB as the total number of mutations present in more than 5% allele frequency [49,50]. Previous reports demonstrated significant correlations in measuring TMB between using WES and these panel-based analyses [23,26,29,51,52], although some studies suggested a certain panel size as the minimum requirement for a correct estimation [51,53]. In our study, the impact of TMB on OS was statistically significant in studies that used NGS but not in studies that used WES. 

In our study, subgroup analyses demonstrated that TMB measurement using panel-based sequencing was associated with a higher OS benefit compared to detection of TMB by WES with moderate heterogeneity. In clinical settings, cost is an important issue for the wide-spread use of diagnostic tools. Consequently, the number of patients using the panel-based sequencing was higher than that of WES-based analysis, and one study, which included an exceptionally large number of patients, used panel-based sequencing, and this might have influenced the results of our meta-analysis [32]. In addition, studies with panel-based NGS sequencing may select the cut off using the optimal value that could maximize the survival difference between patients with high and low TMB. These characteristics of the included studies in our meta-analysis might have caused bias to favor outcomes from panel-based sequencing tests over WES-driven data, rather than one method being superior than the other. Previous studies have reported high correlation of TMB measured by NGS and WES [39,54]. 

Our data also showed that the effect of ICIs on OS was higher in TMB-high patients treated with PD-L1 inhibitors compared to PD-1 inhibitors or cytotoxic T lymphocyte-associated antigen 4 (CTLA-4) inhibitors. This may be related to differences in the mechanism of action of the different ICIs. For example, PD-1 blocking antibodies inhibit the interaction between PD-L1 and PD-1, but they do not inhibit PD-L1 from interacting with CD80 [55]. In contrast, both these interactions with PD-L1 will be blocked by PD-L1-blocking antibodies, but this does not prevent PD-1 from interacting with PD-L2 [55]. In addition, binding of PD-L1 antibodies to tumor-associated macrophages (TAMs) may trigger macrophage proliferation, survival and activation, triggering macrophage-mediated antitumor activity [56]. The CTLA-4 antibodies have a distinct mechanism of action targeting different receptor interactions from PD-L1/PD-1 inhibitors that may also impact a different phase of the anti-tumor immune response. These mechanistic basics might lead to the differences in the predictive impact of TMB for various ICIs. Further studies are needed to clarify these observed differences in more detail.

Conforti and colleagues [57] reported a difference in immunotherapy efficacy according to patient sex. The HRs for OS in patients treated with immunotherapy compared with controls was 0.72 (95% CI 0.65 to 0.79) in men and 0.86 (95% CI 0.79 to 0.93) in women (*p* = 0.0019) [57]. The reason for the observed sex difference was not clear, but differences in behavioral and lifestyle differences were discussed as causative factors [58]. In contrast, Wallis et al. [59] recently updated an earlier meta-analysis and demonstrated no difference in the efficacy of ICIs according to sex. 

Melanoma and NSCLC have high mutational burdens compared with other tumors [51,60], and this was regarded to be the reason that the efficacy of ICIs is most prominent in these cancers. Interestingly, among 19 different cancer types from The Cancer Genome Atlas (TCGA) dataset, mean TMB was only higher in men than in women for cutaneous melanoma [61]. Considering the favorable prognostic impact of high TMB in our meta-analysis, the reduced efficacy of ICIs in female melanoma patients may originate from the relatively lower TMB levels rather than a true sex difference. It should be noted that even though the overall effect size was not significantly different between men and women in the Wallis and colleagues study [59] (HR 0.75, 95% CI (0.69 to 0.81) in men versus HR 0.77, 95% CI (0.67 to 0.88) in women), a reduced efficacy of immunotherapy in women was still noted in the subgroup analysis of melanoma patients (HR 0.68, 95% CI (0.48 to 0.97) in men versus HR 0.83, 95% CI (0.68 to 1.00) in women). Further large-scale clinical investigations should be conducted to validate the real effect of sex or lifestyle factors on immunotherapy efficacy in relation to gender-specific differences in TMB.

Clarifying the association of TMB with other known predictors of ICI therapy may be useful. Association of microsatellite instability and TMB is reported to be complex and differs across different cancer types [62], while PD-L1 expression is known to predict outcome independently from TMB [63,64]. The association of TMB with other clinicopathologic variables known to effect response to ICI therapy such as age, body mass index [65], concomitant medications, gut microbiota [66], mismatch repair status, tumor-infiltrating lymphocytes and neutrophil-to-lymphocyte ratio [1] remains to be elucidated. Recent studies have also demonstrated that genetic driver events, intratumoral heterogeneity, mutational signature and T-cell inflamed gene expression profile may be used to identify patients showing responses to ICIs [30,67,68]. Though several of these factors may be interrelated, these findings suggest that TMB status alone may be insufficient in determining which patients should be offered ICIs. Besides adequate and clinically adapted cut-off values based on the patients’ stage or clinical situation, we suggest a combined approach of these various biomarkers to be evaluated together, as the clinical challenge remains to define non-responders rather than responders. This goal to discriminate non-responders rather than responders should be taken into consideration when defining cut-off values in the cohort of recurrent or advanced disease, as demonstrated by our eligible studies. This might differ for early-stage patients, where a cut-off should identify high-risk patients with the highest probability to benefit from ICI treatment compared to other treatment strategies. Therefore, we suggest a combined approach utilizing a few predictive markers in the future dividing the patient population by subgroups and assessing survival outcomes separately. This may particularly be relevant when the biomarkers are independently predictive, such as PD-L1 and TMB [63,64]. To take account of the combination of many biomarkers to predict response to therapy, one may develop multivariable prediction models (such as logistic regression models) [69,70] or scoring systems [71] which should be followed by its prospective validation in other independent cohorts. Finally, proposed methods should be validated in large-sized RCTs to be utilized in real-world clinics.

There are some remaining issues and limitations in the use of TMB in routine clinical practice. First, even though our study demonstrated that a high TMB level could be interpreted as a positive predictive factor in patients treated with ICIs, deciding the exact cut-off value defining a high TMB level remains a considerable task that should be completed prior to its use in clinical practice. In our eligible studies, cut-off values varied significantly, even across studies of patient groups with the same malignancies. The tendency for HR to become lower as the threshold defining the high TMB increased was observed in several studies [26,27,32]. It may be advantageous to raise the TMB standard to identify patients who will benefit most from ICI treatment, though this may also result in potential responding patients being excluded from ICI therapy. In future studies, an optimal TMB threshold may initially be identified by setting a cut-off of optimal sensitivity and specificity of survival and by utilizing molecular indicators of response to ICI therapy in a discovery cohort, following its prospective validation in another independent cohort [22,72]. The proposed threshold should then be validated in large-sized RCTs to be routinely used in clinical practice.

Another issue regarding TMB as a predictive biomarker is its potential variation over time. For example, TMB may differ with age: Children’s mutational burden of glioblastoma was lower than that of adults [51] and was also changed within the same patient. Nathanson and colleagues [73] reported that TMB was significantly higher in melanoma patients with clinical benefit than in non-responders. However, this positive correlation was only observed when TMB evaluation was performed on patient samples collected prior to treatment and was not maintained when using patient samples collected after the initiation of ICI therapy [73]. Thus, it was suggested that it would be ideal to have TMB assessment on tissue obtained immediately prior to therapy [27]. In our meta-analysis, it was not possible to compare the efficacy of TMB according to the time point at which it was assessed, because many included studies did not report the exact time point at which TMB was assessed.

This study has several limitations. The included studies are apt to have non-generalizable results and may be more representative of an insured, high-income population due to selection and referral bias. Many eligible studies (especially basic research papers) had small sample sizes and often did not present data necessary for analysis, possibly leading to selection bias and exclusion of other meaningful findings. Most eligible studies of high TMB versus low TMB analyses studied NSCLC, melanoma or non-specific tumor type and, therefore, the results may be hard to generalize to other cancer types. Modality of TMB detection (NGS versus WES) and tissue (blood versus tumor) also differed significantly. Definition of high and low TMB varied across the studies, further contributing to the potential heterogeneity. However, the observed heterogeneity (I^2^) within the main analyses or within most subgroups were low with consistent results found across the studies, supporting the robust association of higher benefit of ICIs in the high TMB group compared to the low TMB group despite these differences. Therefore, TMB may be an effective biomarker to predict survival in patients undergoing ICI treatment.

## 4. Materials and Methods

This systematic review and meta-analysis were performed according to Preferred Reporting Items for Systematic Reviews and Meta-Analyses (PRISMA) guidelines [74] (Table A1).

### 4.1. Literature Search Strategy and Eligibility Criteria

Three investigators (JYK, JK and JIS) searched PubMed, Embase, CENTRAL and clinicaltrials.gov from inception to 6 August 2019 to identify retrospective studies or clinical trials of ICIs that reported HRs for OS and/or PFS according to TMB. The search was performed with key words such as tumor mutational burden, mutational load and immune checkpoint inhibitors (full search strategy available in Appendix B). There was no language restriction. Reference lists of relevant studies were also manually searched.

We searched for observational studies or RCTs studying the role of TMB as the predictive biomarker of ICI therapy for cancer patients. The eligibility criteria were as follows. First, the study had to report patient group(s) with any type of cancer treated with inhibitors of PD-1 (nivolumab, pembrolizumab, or toripalimab), PD-L1 (atezolizumab or avelumab), or CTLA-4 (ipilimumab or tremelimumab), alone or in combination with other ICIs. Second, the TMB of the included patients had to be assessed and reported. TMB was assessed by WES or a hybrid capture-based targeted NGS panel, both of which are available in clinical practice. Third, studies were required to either report OS and/or PFS comparing patient groups with high and low TMB or report OS and/or PFS comparing patient groups with ICI treatment and chemotherapy (any kind of chemotherapy) within patient groups with either high or low TMB. The definition of high or low TMB followed that of the individual studies. Fourth, the survival data had to be reported as a calculable metric such as HR. Studies that did not report survival as a calculable metric, in vitro studies, animal studies, conference abstracts and reviews were excluded. When there was a duplicate population among two or more studies, we included the more recent and complete study.

### 4.2. Data Extraction

Three investigators (JYK, JK and JIS) extracted the data, and discrepancies were resolved by discussion and consensus. From eligible articles, we extracted the following: Name of the first author, published year, study design, type of cancer, treatment regimen, source of the sample for assessing TMB, TMB detection method, median TMB and its range, TMB threshold, the numbers of patients in high/low TMB groups and survival outcomes represented as HRs and corresponding 95% CIs.

### 4.3. Statistical Analysis

We performed the following meta-analyses: (1) OS or PFS in the high TMB group versus low TMB group and (2) OS or PFS in ICI arm versus chemotherapy arm within the high TMB group or low TMB group. All analyses were conducted using R version 3.5.1 (R-project, Institute for Statistics and Mathematics) and “metafor” package [75]. We calculated the summary effect size, 95% CI and *p*-values under random- and fixed-effects models. We presented results under random effects because the clinical settings were expected to be heterogeneous across the studies. The heterogeneity among the included studies was evaluated using the I^2^ statistic and its *p*-value of χ2-based Cochran’s Q test. I^2^ > 50% and >75% are considered to indicate large and very large heterogeneity, respectively [76]. To investigate the potential source of variation of the predictive value of TMB, we performed subgroup analyses of treatment regimen, cancer type, TMB sample source (tumor tissue versus blood), TMB detection method (WES versus NGS), study design and the number of participants (<100 versus >100). We assessed publication bias by visual inspection of funnel plots and Egger’s test of asymmetry [77]. In Egger’s test, publication bias was claimed at *p* < 0.10. All statistical tests were two-tailed, and *p* < 0.05 was considered statistically significant.

## 5. Conclusions

Our systematic review demonstrated that the TMB may predict long-term outcomes in patients who received ICIs and encourages its evaluation as a predictive marker of ICI therapy. Future large RCTs of ICIs for patients with various cancer types should evaluate the role of TMB in identifying patient groups that may benefit from ICIs.

## Figures and Tables

**Table 1:** Characteristics of studies included in the meta-analysis of the high tumor mutational burden (TMB) group versus low TMB group.

Study	Type of Study	Malignancy	Type of Immunotherapy	Sample Source	Detection Method	TMB Cutoff	Median TMB (range)	Number of Patients (High/Low TMB)	Outcome
Balar et al. 2017 [44]	Retrospective analysis of clinical trial	Urothelial carcinoma	Atezolizumab	Tumor	FoundationOne	≥16/MB	8.1 (0.9–62.2)	97 (NR)	OS
Chae et al. 2018 [28]	Retrospective cohort	NSCLC	PD-1/PD-L1 inhibitor	Tumor	FoundationOne	≥15/MB	8 (1–55)	34 (NR)	OS, PFS
Chae et al. 2019a [41]	Retrospective cohort	NSCLC	Immune checkpoint inhibitors	Blood	Guardant360	NR (median)	NR	20 (10/10)	OS, PFS
Chae et al. 2019b [41]	Retrospective cohort	NSCLC	Immune checkpoint inhibitors	Blood	Guardant360	NR (median)	NR	12 (6/6)	OS, PFS
Cristescu et al. 2018a [30]	Retrospective analysis of clinical trial	Pan-tumor	Pembrolizumab	Tumor	WES	>102.5	NR	119 (37/82)	PFS
Cristescu et al. 2018b [30]	Retrospective analysis of clinical trial	Melanoma	Pembrolizumab	Tumor	WES	>191.5	NR	89 (59/30)	PFS
Cristescu et al. 2018c [30]	Retrospective analysis of clinical trial	HNSCC	Pembrolizumab	Tumor	WES	>86	NR	107 (54/53)	PFS
Fang et al. 2019 [39]	Retrospective analysis of clinical trial	NSCLC	PD-1/PD-L1 inhibitor	Tumor	WES	≥157 (top tertile)	87 (4–1528)	73 (25/48)	PFS
Goodman et al. 2017 [27]	Retrospective cohort	Various	Various	Tumor	FoundationOne	≥20/MB	6 (1–347)	151 (38/113)	OS, PFS
Hamid et al. 2019 [37]	Retrospective analysis of clinical trial	Melanoma	Atezolizumab	Tumor	FoundationOne	≥16/MB	NR	23 (12/11)	OS, PFS
Hellmann et al. 2018 [23]	Retrospective analysis of clinical trial	NSCLC	Nivolumab plus ipilimumab	Tumor	WES	>158 (median)	158	75 (37/38)	PFS
Hugo et al. 2016 [31]	Retrospective cohort	Melanoma	Pembrolizumab or nivolumab	Tumor	WES	≥577 (bottom tertile)	489 (73–3985)	37 (13/24)	OS
Johnson et al. 2016 [29]	Retrospective cohort	Melanoma	PD-1/PD-L1 inhibitor	Tumor	FoundationOne	>23.1/MB	NR	65 (27/38)	OS, PFS
Khagi et al. 2017 [43]	Retrospective cohort	Various	Various	Blood	Guardant360	>3 total ctDNR alterations	2 (0–20)	69 (20/49)	OS, PFS
Le et al. 2015 [5]	Clinical trial	Various	Pembrolizumab	Tumor	WES	NR	NR	15 (NR)	OS, PFS
Ricciuti et al. 2019 [40]	Retrospective cohort	Small-cell lung cancer	Immune checkpoint inhibitors	Tumor	NGS (OncoPanel)	>9.7/MB (median)	9.8 (1.2–31.2)	52 (26/26)	OS, PFS
Rizvi et al. 2015a [22]	Retrospective cohort	NSCLC	Pembrolizumab	Tumor	WES	>209 (median)	NR	18 (9/9)	PFS
Rizvi et al. 2015b [22]	Retrospective cohort	NSCLC	Pembrolizumab	Tumor	WES	>200 (median)	NR	16 (8/8)	PFS
Rizvi et al. 2018 [26]	Retrospective cohort	NSCLC	Immune checkpoint inhibitors	Tumor	WES	>324	171 (1–1147)	49 (12/37)	PFS
Roszik et al. 2016 [34]	Retrospective cohort	Melanoma	Ipilimumab	Tumor	NGS	>100	NR	76 (57/19)	OS
Samstein et al. 2019 [32]	Retrospective cohort	Various	Immune checkpoint inhibitors	Tumor	NGS (MSK-IMPACT)	90th percentile of each histology	NR	1662 (NR)	OS
Snyder, et al. 2014a [20]	Retrospective cohort	Melanoma	Ipilimumab or tremelimumab	Tumor	WES	>100	NR	25 (10/15)	OS
Snyder et al. 2014b [20]	Retrospective cohort	Melanoma	Ipilimumab or tremelimumab	Tumor	WES	>100	NR	39 (17/22)	OS
Van Allen et al. 2015 [21]	Retrospective cohort	Melanoma	Ipilimumab	Tumor	WES	≥202 (median)	197 (7–5854)	110 (55/55)	OS, PFS
Wang et al. 2019 [38]	Retrospective analysis of clinical trial	Gastric cancer	Toripalimab	Tumor	WES	≥12/MB	NR	54 (12/42)	OS, PFS
Yusko et al. 2019a [35]	Retrospective analysis of clinical trial	Melanoma	Nivolumab or ipilimumab	Tumor	WES	NR	171	30 (NR)	OS
Yusko et al. 2019b [35]	Retrospective analysis of clinical trial	Melanoma	Nivolumab or ipilimumab	Tumor	WES	NR	159	38 (NR)	OS

Abbreviations: TMB, tumor mutational burden; NSCLC, non-small cell lung cancer; HNSCC, head and neck squamous cell carcinoma; PD-1, programmed cell death protein 1; PD-L1, programmed death-ligand 1; WES, whole-exome sequencing; NGS, next-generation sequencing; NR, not reported; OS, overall survival; PFS, progression-free survival.

**Table 2:** Characteristics of studies included in the meta-analysis of the immunotherapy group versus chemotherapy group.

Study	Type of Study	Malignancy	Immunotherapy versus Chemotherapy Comparison	Sample Source	Detection Method	TMB Cutoff	Number of Patients with High/Low TMB	Outcome
Carbone et al. 2017 [33]	Retrospective analysis of RCT	NSCLC	Nivolumab versus platinum-based chemotherapy	Tumor	WES	≥243 (top tertile)	107/205	OS, PFS
Gandara et al. 2018a [42]	Retrospective analysis of RCT	NSCLC	Atezolizumab versus docetaxel	Blood	FoundationOne	≥16/MB	63/148	OS, PFS
Gandara et al. 2018b [42]	Retrospective analysis of RCT	NSCLC	Atezolizumab versus docetaxel	Blood	FoundationOne	≥16/MB	158/425	OS, PFS
Hellmann et al. 2019 * [45]	RCT	NSCLC	Nivolumab plus ipilimumab versus platinum doublet chemotherapy	Tumor	FoundationOne	≥10/MB	299/380	OS
Hellmann et al. 2018a * [25]	RCT	NSCLC	Nivolumab plus ipilimumab versus platinum doublet chemotherapy	Tumor	FoundationOne	≥10/MB	299/380	PFS
Hellmann et al. 2018b [25]	RCT	NSCLC	Nivolumab versus platinum doublet chemotherapy	Tumor	FoundationOne	≥13/MB	150/78	PFS
Powles et al. 2018 [36]	RCT	Urothelial carcinoma	Atezolizumab versus platinum-based chemotherapy	Blood	FoundationOne	≥9.65/MB (median)	274/270	OS

Abbreviations: NR, not reported; NSCLC, non-small cell lung cancer; OS, overall survival; PFS, progression-free survival; RCT, randomized controlled trial; TMB, tumor mutational burden; WES, whole-exome sequencing. * Data from identical population.

**Table 3:** Results of the subgroup analysis of the high TMB group versus low TMB group.

Subgroup	Overall Survival					Progression-Free Survival				
	Number of Study Estimates	HR (95% CI)	*p*-Value *	I^2^ (%)	I^2^ among Subgroups (%)	Number of Study Estimates	HR (95% CI)	*p*-Value *	I^2^ (%)	I^2^ among Subgroups (%)
All studies	19	0.53 (0.42 to 0.67)	<0.001	0		19	0.52 (0.40 to 0.67)	<0.001	0	
Subgroup analysis										
Treatment					0					-
PD-1/PD-L1 inhibitors	7	0.43 (0.29 to 0.64)	<0.001	0		11	0.51 (0.35 to 0.73)	<0.001	0	
CTLA-4 inhibitors	4	0.57 (0.30 to 1.09)	0.087	0						
PD-1 inhibitors versus PD-L1 inhibitors					44					-
PD-1 inhibitors	3	0.62 (0.33 to 1.17)	0.14	0		7	0.54 (0.36 to 0.81)	0.003	0	
PD-L1 inhibitors	2	0.35 (0.21 to 0.61)	<0.001	0						
Cancer type					0					0
Melanoma	9	0.66 (0.43 to 1.01)	0.056	0		4	0.47 (0.21 to 1.05)	0.066	32	
NSCLC	3	1.80 (0.21 to 15.60)	0.59	19		8	0.53 (0.30 to 0.93)	0.028	0	
Sample source					0					0
Tumor tissue	16	0.52 (0.41 to 0.66)	<0.001	0		16	0.50 (0.38 to 0.66)	<0.001	0	
Blood	3	1.22 (0.21 to 7.21)	0.83	39		3	0.84 (0.26 to 2.70)	0.77	18	
Detection method					77					0
WES	8	0.73 (0.50 to 1.06)	0.094	0		11	0.56 (0.41 to 0.77)	<0.001	0	
NGS	11	0.44 (0.33 to 0.59)	<0.001	0		8	0.44 (0.26 to 0.73)	0.001	6	
Data source					0					0
Clinical trials	6	0.57 (0.35 to 0.92)	0.020	32		8	0.52 (0.36 to 0.75)	<0.001	0	
Cohorts	13	0.50 (0.37 to 0.68)	<0.001	0		11	0.51 (0.35 to 0.76)	<0.001	1	
Number of participants					0					0
≥100 participants	3	0.53 (0.37 to 0.75)	<0.001	0		4	0.56 (0.37 to 0.85)	0.007	7	
<100 participants	16	0.53 (0.39 to 0.72)	<0.001	0		15	0.49 (0.34 to 0.69)	<0.001	0	

Abbreviations: CI, confidence interval; CTLA-4, cytotoxic T lymphocyte-associated antigen 4; HR, hazard ratio; NGS, next-generation sequencing; NSCLC, non-small cell lung cancer; PD-1, programmed cell death protein 1; PD-L1, programmed death-ligand 1; WES, whole-exome sequencing. * Significant associations are shown in bold.

## Supplementary Materials

The following are available online at https://www.mdpi.com/2072-6694/11/11/1798/s1, Table S1: Results of random-effects, fixed-effects, heterogeneity and Egger *p*-value of all meta-analyses, Figure S1: Overall survival, high TMB versus low TMB, funnel plot, Figure S2: Progression-free survival, high TMB versus low TMB, funnel plot, Figure S3: Overall survival, high TMB, immunotherapy versus chemotherapy, random-effects model, Figure S4: Progression-free survival, high TMB, immunotherapy versus chemotherapy, random-effects model, Figure S5: Overall survival, low TMB, immunotherapy versus chemotherapy, random-effects model, Figure S6: Progression-free survival, low TMB, immunotherapy versus chemotherapy, random-effects model, Figure S7: Overall survival, high TMB, immunotherapy versus chemotherapy, funnel plot, Figure S8: Progression-free survival, high TMB, immunotherapy versus chemotherapy, funnel plot, Figure S9: Overall survival, low TMB, immunotherapy versus chemotherapy, funnel plot, Figure S10: Progression-free survival, low TMB, immunotherapy versus chemotherapy, funnel plot.

## Appendix A

**Table A1:** PRISMA Checklist.

Title	1	Identify the Report as a Systematic Review, Meta-Analysis, or Both	0
ABSTRACT			
Structured summary	2	Provide a structured summary including, as applicable: Background; objectives; data sources; study eligibility criteria, participants, and interventions; study appraisal and synthesis methods; results; limitations; conclusions and implications of key findings; systematic review registration number.	0
INTRODUCTION			
Rationale	3	Describe the rationale for the review in the context of what is already known.	1
Objectives	4	Provide an explicit statement of questions being addressed with reference to participants, interventions, comparisons, outcomes, and study design (PICOS).	1
METHODS			
Protocol and registration	5	Indicate if a review protocol exists, if and where it can be accessed (e.g., Web address), and, if available, provide registration information including registration number.	N/A
Eligibility criteria	6	Specify study characteristics (e.g., PICOS, length of follow-up) and report characteristics (e.g., years considered, language, publication status) used as criteria for eligibility, giving rationale.	11–12
Information sources	7	Describe all information sources (e.g., databases with dates of coverage, contact with study authors to identify additional studies) in the search and date last searched.	11
Search	8	Present full electronic search strategy for at least one database, including any limits used, such that it could be repeated.	Appendix B
Study selection	9	State the process for selecting studies (i.e., screening, eligibility, included in systematic review, and, if applicable, included in the meta-analysis).	11–12
Data collection process	10	Describe method of data extraction from reports (e.g., piloted forms, independently, in duplicate) and any processes for obtaining and confirming data from investigators.	12
Data items	11	List and define all variables for which data were sought (e.g., PICOS, funding sources) and any assumptions and simplifications made.	12
Risk of bias in individual studies	12	Describe methods used for assessing risk of bias of individual studies (including specification of whether this was done at the study or outcome level), and how this information is to be used in any data synthesis.	N/A
Summary measures	13	State the principal summary measures (e.g., risk ratio, difference in means).	12
Synthesis of results	14	Describe the methods of handling data and combining results of studies, if done, including measures of consistency (e.g., I^2^) for each meta-analysis.	12
Risk of bias across studies	15	Specify any assessment of risk of bias that may affect the cumulative evidence (e.g., publication bias, selective reporting within studies).	12
Additional analyses	16	Describe methods of additional analyses (e.g., sensitivity or subgroup analyses, meta-regression), if done, indicating which were pre-specified.	12
RESULTS			
Study selection	17	Give numbers of studies screened, assessed for eligibility, and included in the review, with reasons for exclusions at each stage, ideally with a flow diagram.	2, Figure 1
Study characteristics	18	For each study, present characteristics for which data were extracted (e.g., study size, PICOS, follow-up period) and provide the citations.	2, Table 1 and Table 2
Risk of bias within studies	19	Present data on risk of bias of each study and, if available, any outcome level assessment (see item 12).	N/A
Results of individual studies	20	For all outcomes considered (benefits or harms), present, for each study: (a) simple summary data for each intervention group (b) effect estimates and confidence intervals, ideally with a forest plot.	Figure 2 and Figure 3, Figures S3–S6
Synthesis of results	21	Present results of each meta-analysis done, including confidence intervals and measures of consistency.	2, Table 1 and Table 2, Table S1
Risk of bias across studies	22	Present results of any assessment of risk of bias across studies (see Item 15).	2, Table S1, Figures S1, S2, S7–S10
Additional analysis	23	Give results of additional analyses, if done (e.g., sensitivity or subgroup analyses, meta-regression [see Item 16]).	Table 3
DISCUSSION			
Summary of evidence	24	Summarize the main findings including the strength of evidence for each main outcome; consider their relevance to key groups (e.g., healthcare providers, users, and policy makers).	9–11
Limitations	25	Discuss limitations at study and outcome level (e.g., risk of bias), and at review-level (e.g., incomplete retrieval of identified research, reporting bias).	11
Conclusions	26	Provide a general interpretation of the results in the context of other evidence, and implications for future research.	12
FUNDING			
Funding	27	Describe sources of funding for the systematic review and other support (e.g., supply of data); role of funders for the systematic review.	13(none)

From: Moher et al. [74]

## Appendix B

### Full Search Strategy in PubMed

The last search performed on 6 August 2019 yielded 776 results:

Search term: (mutational burden* OR mutation burden* OR mutational load* OR mutation load*) AND (nivolumab OR pembrolizumab OR atezolizumab OR avelumab OR durvalumab OR ipilimumab OR immunotherap* OR PD-1 OR PD-L1 OR CTLA-4 OR PD1 OR PDL1 OR CTLA4 OR PDCD1 OR CD274 OR BMS-936558 OR BMS-936559 OR MK-3475 OR MPDL3280A OR MEDI4736 OR MSB0010718C OR BMS-734016 OR MDX-010 OR MDX-101 OR immune checkpoint* OR checkpoint blockade*)

## References

[1] Gibney G.T., Weiner L.M., Atkins M.B. (2016). Predictive biomarkers for checkpoint inhibitor-based immunotherapy. Lancet Oncol..

[2] Wei S.C., Duffy C.R., Allison J.P. (2018). Fundamental Mechanisms of Immune Checkpoint Blockade Therapy. Cancer Discov..

[3] Robert C., Long G.V., Brady B., Dutriaux C., Maio M., Mortier L., Hassel J.C., Rutkowski P., McNeil C., Kalinka-Warzocha E. (2015). Nivolumab in previously untreated melanoma without BRAF mutation. N. Engl. J. Med..

[4] Brahmer J., Reckamp K.L., Baas P., Crino L., Eberhardt W.E., Poddubskaya E., Antonia S., Pluzanski A., Vokes E.E., Holgado E. (2015). Nivolumab versus Docetaxel in Advanced Squamous-Cell Non-Small-Cell Lung Cancer. N. Engl. J. Med..

[5] Le D.T., Uram J.N., Wang H., Bartlett B.R., Kemberling H., Eyring A.D., Skora A.D., Luber B.S., Azad N.S., Laheru D. (2015). PD-1 Blockade in Tumors with Mismatch-Repair Deficiency. N. Engl. J. Med..

[6] Hansen A.R., Siu L.L. (2016). PD-L1 Testing in Cancer: Challenges in Companion Diagnostic Development. JAMA Oncol..

[7] Reck M., Rodriguez-Abreu D., Robinson A.G., Hui R., Csoszi T., Fulop A., Gottfried M., Peled N., Tafreshi A., Cuffe S. (2016). Pembrolizumab versus Chemotherapy for PD-L1-Positive Non-Small-Cell Lung Cancer. N. Engl. J. Med..

[8] Shen X., Zhao B. (2018). Efficacy of PD-1 or PD-L1 inhibitors and PD-L1 expression status in cancer: Meta-analysis. BMJ.

[9] Brody R., Zhang Y., Ballas M., Siddiqui M.K., Gupta P., Barker C., Midha A., Walker J. (2017). PD-L1 expression in advanced NSCLC: Insights into risk stratification and treatment selection from a systematic literature review. Lung Cancer.

[10] Buttner R., Gosney J.R., Skov B.G., Adam J., Motoi N., Bloom K.J., Dietel M., Longshore J.W., Lopez-Rios F., Penault-Llorca F. (2017). Programmed Death-Ligand 1 Immunohistochemistry Testing: A Review of Analytical Assays and Clinical Implementation in Non-Small-Cell Lung Cancer. J. Clin. Oncol..

[11] Rimm D.L., Han G., Taube J.M., Yi E.S., Bridge J.A., Flieder D.B., Homer R., West W.W., Wu H., Roden A.C. (2017). A Prospective, Multi-institutional, Pathologist-Based Assessment of 4 Immunohistochemistry Assays for PD-L1 Expression in Non-Small Cell Lung Cancer. JAMA Oncol..

[12] Tumeh P.C., Harview C.L., Yearley J.H., Shintaku I.P., Taylor E.J., Robert L., Chmielowski B., Spasic M., Henry G., Ciobanu V. (2014). PD-1 blockade induces responses by inhibiting adaptive immune resistance. Nature.

[13] Horn L., Spigel D.R., Vokes E.E., Holgado E., Ready N., Steins M., Poddubskaya E., Borghaei H., Felip E., Paz-Ares L. (2017). Nivolumab Versus Docetaxel in Previously Treated Patients With Advanced Non-Small-Cell Lung Cancer: Two-Year Outcomes From Two Randomized, Open-Label, Phase III Trials (CheckMate 017 and CheckMate 057). J. Clin. Oncol..

[14] Motzer R.J., Escudier B., McDermott D.F., George S., Hammers H.J., Srinivas S., Tykodi S.S., Sosman J.A., Procopio G., Plimack E.R. (2015). Nivolumab versus Everolimus in Advanced Renal-Cell Carcinoma. N. Engl. J. Med..

[15] Rittmeyer A., Barlesi F., Waterkamp D., Park K., Ciardiello F., von Pawel J., Gadgeel S.M., Hida T., Kowalski D.M., Dols M.C. (2017). Atezolizumab versus docetaxel in patients with previously treated non-small-cell lung cancer (OAK): A phase 3, open-label, multicentre randomised controlled trial. Lancet.

[16] Topalian S.L., Taube J.M., Anders R.A., Pardoll D.M. (2016). Mechanism-driven biomarkers to guide immune checkpoint blockade in cancer therapy. Nat. Rev. Cancer.

[17] Wang J., Chmielowski B., Pellissier J., Xu R., Stevinson K., Liu F.X. (2017). Cost-Effectiveness of Pembrolizumab Versus Ipilimumab in Ipilimumab-Naive Patients with Advanced Melanoma in the United States. J. Manag. Care Spec. Pharm..

[18] Oh A., Tran D.M., McDowell L.C., Keyvani D., Barcelon J.A., Merino O., Wilson L. (2017). Cost-Effectiveness of Nivolumab-Ipilimumab Combination Therapy Compared with Monotherapy for First-Line Treatment of Metastatic Melanoma in the United States. J. Manag. Care Spec. Pharm..

[19] Khoja L., Day D., Wei-Wu Chen T., Siu L.L., Hansen A.R. (2017). Tumour- and class-specific patterns of immune-related adverse events of immune checkpoint inhibitors: A systematic review. Ann. Oncol..

[20] Snyder A., Makarov V., Merghoub T., Yuan J., Zaretsky J.M., Desrichard A., Walsh L.A., Postow M.A., Wong P., Ho T.S. (2014). Genetic basis for clinical response to CTLA-4 blockade in melanoma. N. Engl. J. Med..

[21] Van Allen E.M., Miao D., Schilling B., Shukla S.A., Blank C., Zimmer L., Sucker A., Hillen U., Foppen M.H.G., Goldinger S.M. (2015). Genomic correlates of response to CTLA-4 blockade in metastatic melanoma. Science.

[22] Rizvi N.A., Hellmann M.D., Snyder A., Kvistborg P., Makarov V., Havel J.J., Lee W., Yuan J., Wong P., Ho T.S. (2015). Cancer immunology. Mutational landscape determines sensitivity to PD-1 blockade in non-small cell lung cancer. Science.

[23] Hellmann M.D., Nathanson T., Rizvi H., Creelan B.C., Sanchez-Vega F., Ahuja A., Ni A., Novik J.B., Mangarin L.M.B., Abu-Akeel M. (2018). Genomic Features of Response to Combination Immunotherapy in Patients with Advanced Non-Small-Cell Lung Cancer. Cancer Cell.

[24] Yarchoan M., Hopkins A., Jaffee E.M. (2017). Tumor Mutational Burden and Response Rate to PD-1 Inhibition. N. Engl. J. Med..

[25] Hellmann M.D., Ciuleanu T.E., Pluzanski A., Lee J.S., Otterson G.A., Audigier-Valette C., Minenza E., Linardou H., Burgers S., Salman P. (2018). Nivolumab plus Ipilimumab in Lung Cancer with a High Tumor Mutational Burden. N. Engl. J. Med..

[26] Rizvi H., Sanchez-Vega F., La K., Chatila W., Jonsson P., Halpenny D., Plodkowski A., Long N., Sauter J.L., Rekhtman N. (2018). Molecular Determinants of Response to Anti-Programmed Cell Death (PD)-1 and Anti-Programmed Death-Ligand 1 (PD-L1) Blockade in Patients With Non-Small-Cell Lung Cancer Profiled With Targeted Next-Generation Sequencing. J. Clin. Oncol..

[27] Goodman A.M., Kato S., Bazhenova L., Patel S.P., Frampton G.M., Miller V., Stephens P.J., Daniels G.A., Kurzrock R. (2017). Tumor Mutational Burden as an Independent Predictor of Response to Immunotherapy in Diverse Cancers. Mol. Cancer.

[28] Chae Y.K., Davis A.A., Raparia K., Agte S., Pan A., Mohindra N., Villaflor V., Giles F. (2018). Association of Tumor Mutational Burden with DNA Repair Mutations and Response to Anti-PD-1/PD-L1 Therapy in Non-Small-Cell Lung Cancer. Clin. Lung Cancer.

[29] Johnson D.B., Frampton G.M., Rioth M.J., Yusko E., Xu Y., Guo X., Ennis R.C., Fabrizio D., Chalmers Z.R., Greenbowe J. (2016). Targeted Next Generation Sequencing Identifies Markers of Response to PD-1 Blockade. Cancer Immunol. Res..

[30] Cristescu R., Mogg R., Ayers M., Albright A., Murphy E., Yearley J., Sher X., Liu X.Q., Lu H., Nebozhyn M. (2018). Pan-tumor genomic biomarkers for PD-1 checkpoint blockade-based immunotherapy. Science.

[31] Hugo W., Zaretsky J.M., Sun L., Song C., Moreno B.H., Hu-Lieskovan S., Berent-Maoz B., Pang J., Chmielowski B., Cherry G. (2016). Genomic and Transcriptomic Features of Response to Anti-PD-1 Therapy in Metastatic Melanoma. Cell.

[32] Samstein R.M., Lee C.H., Shoushtari A.N., Hellmann M.D., Shen R., Janjigian Y.Y., Barron D.A., Zehir A., Jordan E.J., Omuro A. (2019). Tumor mutational load predicts survival after immunotherapy across multiple cancer types. Nat. Genet..

[33] Carbone D.P., Reck M., Paz-Ares L., Creelan B., Horn L., Steins M., Felip E., van den Heuvel M.M., Ciuleanu T.E., Badin F. (2017). First-Line Nivolumab in Stage IV or Recurrent Non-Small-Cell Lung Cancer. N. Engl. J. Med..

[34] Roszik J., Haydu L.E., Hess K.R., Oba J., Joon A.Y., Siroy A.E., Karpinets T.V., Stingo F.C., Baladandayuthapani V., Tetzlaff M.T. (2016). Novel algorithmic approach predicts tumor mutation load and correlates with immunotherapy clinical outcomes using a defined gene mutation set. BMC Med..

[35] Yusko E., Vignali M., Wilson R.K., Mardis E.R., Hodi F.S., Horak C.E., Chang H., Woods D., Robins H., Weber J.S. (2019). Association of Tumor Microenvironment T-Cell Repertoire and Mutational Load With Clinical Outcome After Sequential Checkpoint Blockade in Melanoma. Cancer Immunol. Res..

[36] Powles T., Duran I., van der Heijden M.S., Loriot Y., Vogelzang N.J., De Giorgi U., Oudard S., Retz M.M., Castellano D., Bamias A. (2018). Atezolizumab versus chemotherapy in patients with platinum-treated locally advanced or metastatic urothelial carcinoma (IMvigor211): A multicentre, open-label, phase 3 randomised controlled trial. Lancet.

[37] Hamid O., Molinero L., Bolen C.R., Sosman J.A., Munoz-Couselo E., Kluger H.M., McDermott D.F., Powderly J., Sarkar I., Ballinger M. (2019). Safety, Clinical Activity, and Biological Correlates of Response in Patients With Metastatic Melanoma: Results From a Phase I trial of Atezolizumab. Clin. Cancer Res..

[38] Wang F., Wei X.L., Wang F.H., Xu N., Shen L., Dai G.H., Yuan X.L., Chen Y., Yang S.J., Shi J.H. (2019). Safety, efficacy and tumor mutational burden as a biomarker of overall survival benefit in chemo-refractory gastric cancer treated with toripalimab, a PD1 antibody in phase Ib/II clinical trial NCT02915432. Ann. Oncol..

[39] Fang W., Ma Y., Yin J.C., Hong S., Zhou H., Wang A., Wang F., Bao H., Wu X., Yang Y. (2019). Comprehensive Genomic Profiling Identifies Novel Genetic Predictors of Response to Anti-PD-(L)1 Therapies in Non-Small Cell Lung Cancer. Clin Cancer Res.

[40] Ricciuti B., Kravets S., Dahlberg S.E., Umeton R., Albayrak A., Subegdjo S.J., Johnson B.E., Nishino M., Sholl L.M., Awad M.M. (2019). Use of targeted next generation sequencing to characterize tumor mutational burden and efficacy of immune checkpoint inhibition in small cell lung cancer. J. Immunother. Cancer.

[41] Chae Y.K., Davis A.A., Agte S., Pan A., Simon N.I., Iams W.T., Cruz M.R., Tamragouri K., Rhee K., Mohindra N. (2019). Clinical Implications of Circulating Tumor DNA Tumor Mutational Burden (ctDNA TMB) in Non-Small Cell Lung Cancer. Oncologist.

[42] Gandara D.R., Paul S.M., Kowanetz M., Schleifman E., Zou W., Li Y., Rittmeyer A., Fehrenbacher L., Otto G., Malboeuf C. (2018). Blood-based tumor mutational burden as a predictor of clinical benefit in non-small-cell lung cancer patients treated with atezolizumab. Nat. Med..

[43] Khagi Y., Goodman A.M., Daniels G.A., Patel S.P., Sacco A.G., Randall J.M., Bazhenova L.A., Kurzrock R. (2017). Hypermutated Circulating Tumor DNA: Correlation with Response to Checkpoint Inhibitor-Based Immunotherapy. Clin. Cancer Res..

[44] Balar A.V., Galsky M.D., Rosenberg J.E., Powles T., Petrylak D.P., Bellmunt J., Loriot Y., Necchi A., Hoffman-Censits J., Perez-Gracia J.L. (2017). Atezolizumab as first-line treatment in cisplatin-ineligible patients with locally advanced and metastatic urothelial carcinoma: A single-arm, multicentre, phase 2 trial. Lancet.

[45] Hellmann M.D., Paz-Ares L., Bernabe Caro R., Zurawski B., Kim S.W., Carcereny Costa E., Park K., Alexandru A., Lupinacci L., de la Mora Jimenez E. (2019). Nivolumab plus Ipilimumab in Advanced Non-Small-Cell Lung Cancer. N. Engl. J. Med..

[46] Cao D., Xu H., Xu X., Guo T., Ge W. (2019). High tumor mutation burden predicts better efficacy of immunotherapy: A pooled analysis of 103078 cancer patients. Oncoimmunology.

[47] Frampton G.M., Fichtenholtz A., Otto G.A., Wang K., Downing S.R., He J., Schnall-Levin M., White J., Sanford E.M., An P. (2013). Development and validation of a clinical cancer genomic profiling test based on massively parallel DNA sequencing. Nat. Biotechnol..

[48] Cheng D.T., Mitchell T.N., Zehir A., Shah R.H., Benayed R., Syed A., Chandramohan R., Liu Z.Y., Won H.H., Scott S.N. (2015). Memorial Sloan Kettering-Integrated Mutation Profiling of Actionable Cancer Targets (MSK-IMPACT): A Hybridization Capture-Based Next-Generation Sequencing Clinical Assay for Solid Tumor Molecular Oncology. J. Mol. Diagn..

[49] Chan T.A., Yarchoan M., Jaffee E., Swanton C., Quezada S.A., Stenzinger A., Peters S. (2019). Development of tumor mutation burden as an immunotherapy biomarker: Utility for the oncology clinic. Ann. Oncol..

[50] Hendriks L.E., Rouleau E., Besse B. (2018). Clinical utility of tumor mutational burden in patients with non-small cell lung cancer treated with immunotherapy. Transl. Lung Cancer Res..

[51] Chalmers Z.R., Connelly C.F., Fabrizio D., Gay L., Ali S.M., Ennis R., Schrock A., Campbell B., Shlien A., Chmielecki J. (2017). Analysis of 100,000 human cancer genomes reveals the landscape of tumor mutational burden. Genome Med..

[52] Rosenberg J.E., Hoffman-Censits J., Powles T., van der Heijden M.S., Balar A.V., Necchi A., Dawson N., O’Donnell P.H., Balmanoukian A., Loriot Y. (2016). Atezolizumab in patients with locally advanced and metastatic urothelial carcinoma who have progressed following treatment with platinum-based chemotherapy: A single-arm, multicentre, phase 2 trial. Lancet.

[53] Buchhalter I., Rempel E., Endris V., Allgauer M., Neumann O., Volckmar A.L., Kirchner M., Leichsenring J., Lier A., von Winterfeld M. (2019). Size matters: Dissecting key parameters for panel-based tumor mutational burden analysis. Int. J. Cancer.

[54] Chang H., Sasson A., Srinivasan S., Golhar R., Greenawalt D.M., Geese W.J., Green G., Zerba K., Kirov S., Szustakowski J. (2019). Bioinformatic Methods and Bridging of Assay Results for Reliable Tumor Mutational Burden Assessment in Non-Small-Cell Lung Cancer. Mol. Diagn..

[55] Alsaab H.O., Sau S., Alzhrani R., Tatiparti K., Bhise K., Kashaw S.K., Iyer A.K. (2017). PD-1 and PD-L1 Checkpoint Signaling Inhibition for Cancer Immunotherapy: Mechanism, Combinations, and Clinical Outcome. Front Pharm..

[56] Hartley G.P., Chow L., Ammons D.T., Wheat W.H., Dow S.W. (2018). Programmed Cell Death Ligand 1 (PD-L1) Signaling Regulates Macrophage Proliferation and Activation. Cancer Immunol. Res..

[57] Conforti F., Pala L., Bagnardi V., De Pas T., Martinetti M., Viale G., Gelber R.D., Goldhirsch A. (2018). Cancer immunotherapy efficacy and patients’ sex: A systematic review and meta-analysis. Lancet Oncol..

[58] Carrera C., Potrony M., Puig S. (2018). Sex as a predictor of response to cancer immunotherapy. Lancet Oncol..

[59] Wallis CJ D., Butaney M., Satkunasivam R., Freedland S.J., Patel S.P., Hamid O., Pal S.K., Klaassen Z. (2019). Association of Patient Sex with Efficacy of Immune Checkpoint Inhibitors and Overall Survival in Advanced Cancers: A Systematic Review and Meta-analysis. JAMA Oncol..

[60] Alexandrov L.B., Nik-Zainal S., Wedge D.C., Aparicio S.A., Behjati S., Biankin A.V., Bignell G.R., Bolli N., Borg A., Borresen-Dale A.L. (2013). Signatures of mutational processes in human cancer. Nature.

[61] Gupta S., Artomov M., Goggins W., Daly M., Tsao H. (2015). Gender Disparity and Mutation Burden in Metastatic Melanoma. J. Natl. Cancer Inst..

[62] Luchini C., Bibeau F., Ligtenberg MJ L., Singh N., Nottegar A., Bosse T., Miller R., Riaz N., Douillard J.Y., Andre F. (2019). ESMO recommendations on microsatellite instability testing for immunotherapy in cancer, and its relationship with PD-1/PD-L1 expression and tumour mutational burden: A systematic review-based approach. Ann. Oncol..

[63] Yu Y., Zeng D., Ou Q., Liu S., Li A., Chen Y., Lin D., Gao Q., Zhou H., Liao W. (2019). Association of Survival and Immune-Related Biomarkers With Immunotherapy in Patients With Non-Small Cell Lung Cancer: A Meta-analysis and Individual Patient-Level Analysis. JAMA Netw. Open.

[64] Yarchoan M., Albacker L.A., Hopkins A.C., Montesion M., Murugesan K., Vithayathil T.T., Zaidi N., Azad N.S., Laheru D.A., Frampton G.M. (2019). PD-L1 expression and tumor mutational burden are independent biomarkers in most cancers. JCI Insight.

[65] McQuade J.L., Daniel C.R., Hess K.R., Mak C., Wang D.Y., Rai R.R., Park J.J., Haydu L.E., Spencer C., Wongchenko M. (2018). Association of body-mass index and outcomes in patients with metastatic melanoma treated with targeted therapy, immunotherapy, or chemotherapy: A retrospective, multicohort analysis. Lancet Oncol..

[66] Pinato D.J., Gramenitskaya D., Altmann D.M., Boyton R.J., Mullish B.H., Marchesi J.R., Bower M. (2019). Antibiotic therapy and outcome from immune-checkpoint inhibitors. J. Immunother. Cancer.

[67] Miao D., Margolis C.A., Vokes N.I., Liu D., Taylor-Weiner A., Wankowicz S.M., Adeegbe D., Keliher D., Schilling B., Tracy A. (2018). Genomic correlates of response to immune checkpoint blockade in microsatellite-stable solid tumors. Nat. Genet..

[68] Conway J.R., Kofman E., Mo S.S., Elmarakeby H., Van Allen E. (2018). Genomics of response to immune checkpoint therapies for cancer: Implications for precision medicine. Genome Med..

[69] Zhai T.T., van Dijk L.V., Huang B.T., Lin Z.X., Ribeiro C.O., Brouwer C.L., Oosting S.F., Halmos G.B., Witjes MJ H., Langendijk J.A. (2017). Improving the prediction of overall survival for head and neck cancer patients using image biomarkers in combination with clinical parameters. Radiother. Oncol..

[70] Beukinga R.J., Hulshoff J.B., Mul VE M., Noordzij W., Kats-Ugurlu G., Slart R., Plukker J.T.M. (2018). Prediction of Response to Neoadjuvant Chemotherapy and Radiation Therapy with Baseline and Restaging (18)F-FDG PET Imaging Biomarkers in Patients with Esophageal Cancer. Radiology.

[71] Hur H., Tulina I., Cho M.S., Min B.S., Koom W.S., Lim J.S., Ahn J.B., Kim N.K. (2016). Biomarker-Based Scoring System for Prediction of Tumor Response After Preoperative Chemoradiotherapy in Rectal Cancer by Reverse Transcriptase Polymerase Chain Reaction Analysis. Dis. Colon Rectum.

[72] Panda A., Betigeri A., Subramanian K., Ross J.S., Pavlick D.C., Ali S., Markowski P., Silk A., Kaufman H.L., Lattime E. (2017). Identifying a Clinically Applicable Mutational Burden Threshold as a Potential Biomarker of Response to Immune Checkpoint Therapy in Solid Tumors. JCO Precis. Oncol..

[73] Nathanson T., Ahuja A., Rubinsteyn A., Aksoy B.A., Hellmann M.D., Miao D., Van Allen E., Merghoub T., Wolchok J.D., Snyder A. (2017). Somatic Mutations and Neoepitope Homology in Melanomas Treated with CTLA-4 Blockade. Cancer Immunol. Res..

[74] Moher D., Liberati A., Tetzlaff J., Altman D. G. (2009). Preferred reporting items for systematic reviews and meta-analyses: The PRISMA statement. BMJ.

[75] Viechtbauer W. (2010). Conducting meta-analyses in R with, the metafor package. J. Stat. Softw..

[76] Higgins J.P., Thompson S.G., Deeks J.J., Altman D.G. (2003). Measuring inconsistency in meta-analyses. BMJ.

[77] Egger M., Davey Smith G., Schneider M., Minder C. (1997). Bias in meta-analysis detected by a simple, graphical test. BMJ.

