# Supplementary material for: Tumor Mutational Burden and Efficacy of Immune Checkpoint Inhibitors: A Systematic Review and Meta-Analysis
Source: Cancers (Basel). 2019 Nov 15;11(11):1798. doi: 10.3390/cancers11111798 (PMC6895916; doi:10.3390/cancers11111798)
Supplement: Supplementary file 1 [file cancers-11-01798-s001.pdf]

Tumor Mutational Burden and Efficacy of Immune Checkpoint Inhibitors: A Systematic Review and Meta-Analysis

Jong Yeob Kim, Andreas Kronbichler, Michael Eisenhut, Sung Hwi Hong, Hans J. van der Vliet, Jeonghyun Kang, Jae Il Shin and Gabriele Gamerith

Table S1. Results of random-effects, fixed-effects, heterogeneity and Egger *p* value of all meta-analyses.

Meta-analysis	Number of Studies	Number of Participants	Random Effects Summary Estimate	Random Effects *p* Value	Fixed Effects Summary Estimate	Fixed Effects *p* Value	I2	Egger *p* Value
Overall survival, high TMB vs low TMB	19	2609	0.53 (0.42 to 0.67)	0.000000053	0.53 (0.42 to 0.67)	0.000000053	0	0.5
Progression-free survival, high TMB vs low TMB	19	1151	0.52 (0.40 to 0.67)	0.0000011	0.52 (0.40 to 0.67)	0.0000011	0	0.48
Overall survival, high TMB, immunotherapy vs chemotherapy	5	2329	0.69 (0.50 to 0.95)	0.023	0.69 (0.50 to 0.95)	0.023	0	0.63
Overall survival, low TMB, immunotherapy vs chemotherapy	4	2118	0.78 (0.60 to 1.00)	0.051	0.78 (0.60 to 1.00)	0.051	0	0.12
Progression-free survival, high TMB, immunotherapy vs chemotherapy	5	2013	0.66 (0.47 to 0.92)	0.014	0.66 (0.47 to 0.92)	0.014	0	0.86
Progression-free survival, low TMB, immunotherapy vs chemotherapy	3	1574	1.14 (0.83 to 1.57)	0.41	1.12 (0.85 to 1.48)	0.4	21	0.097

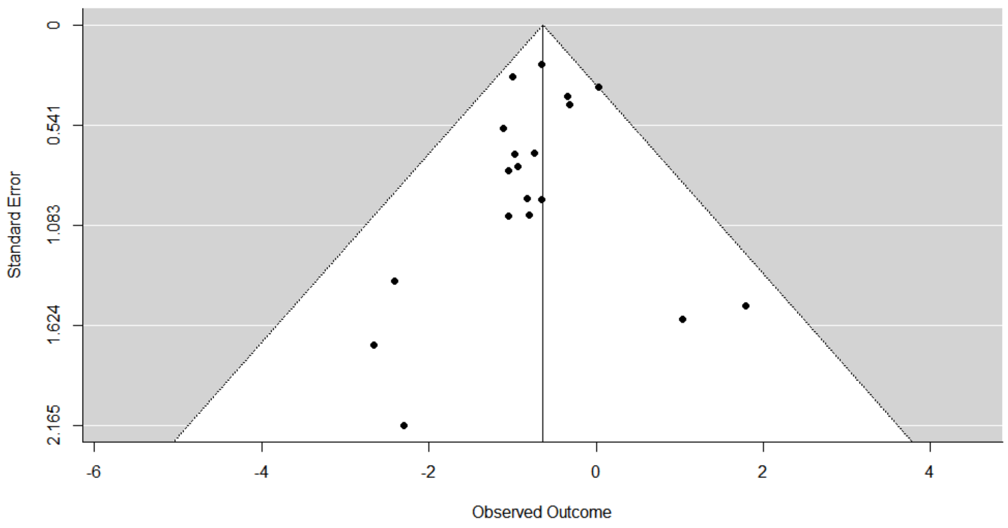

Figure S1. Overall survival, high TMB vs low TMB, funnel plot.

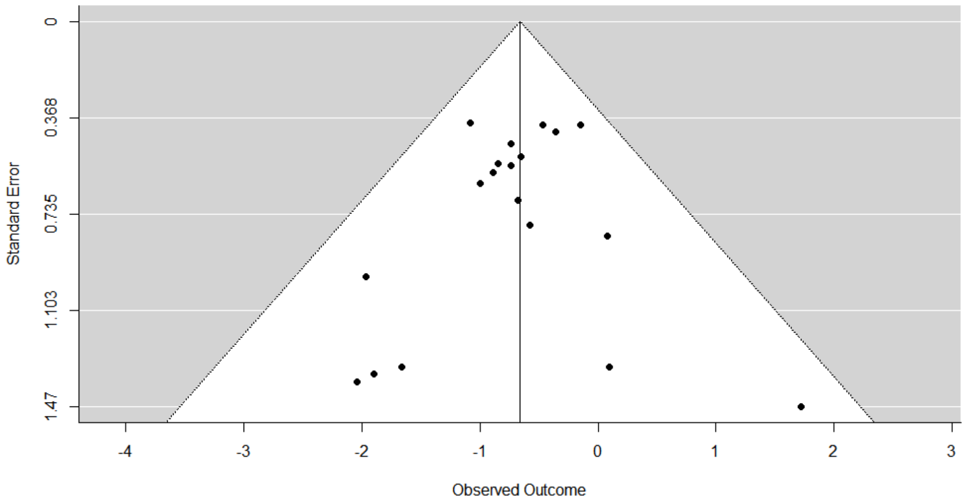

Figure S2. Progression-free survival, high TMB vs low TMB, funnel plot.

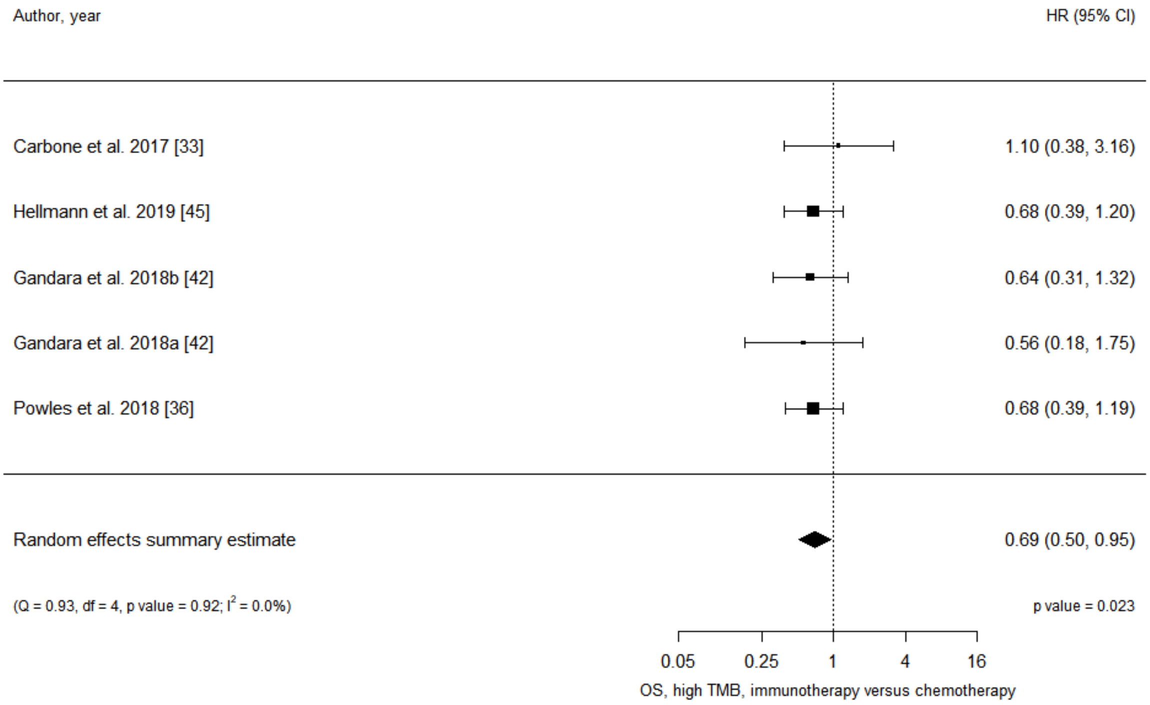

Figure S3. Overall survival, high TMB, immunotherapy vs chemotherapy, random-effects model.

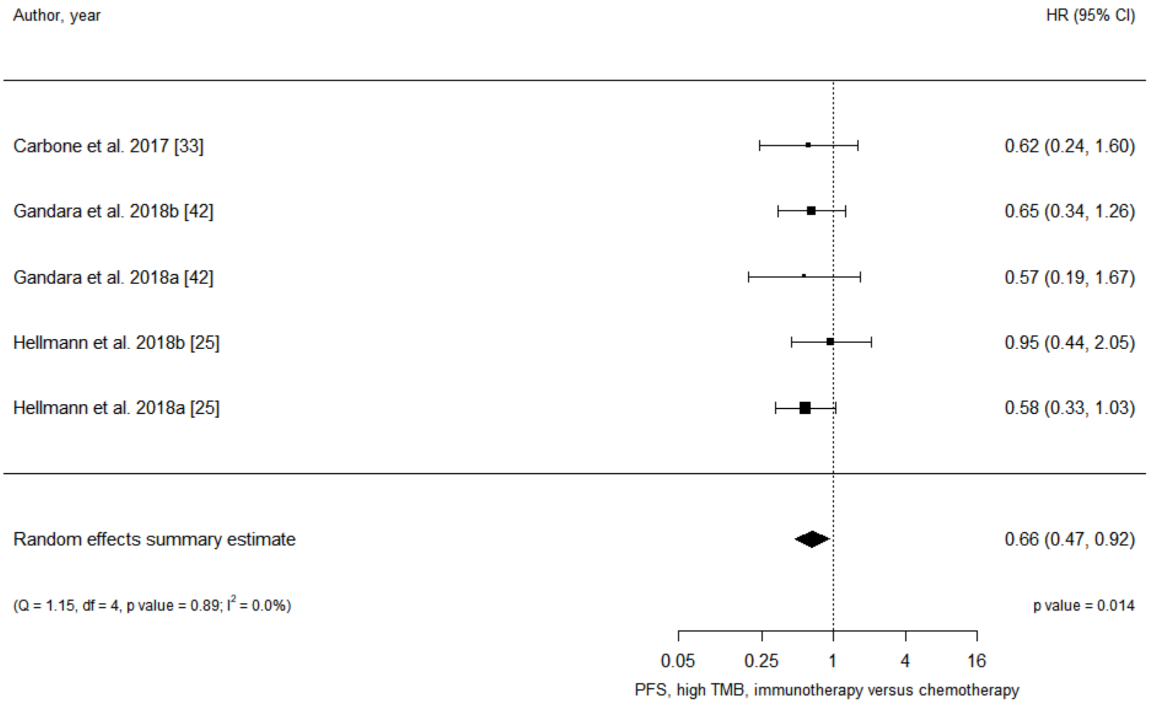

Figure S4. Progression-free survival, high TMB, immunotherapy vs chemotherapy, random-effects model.

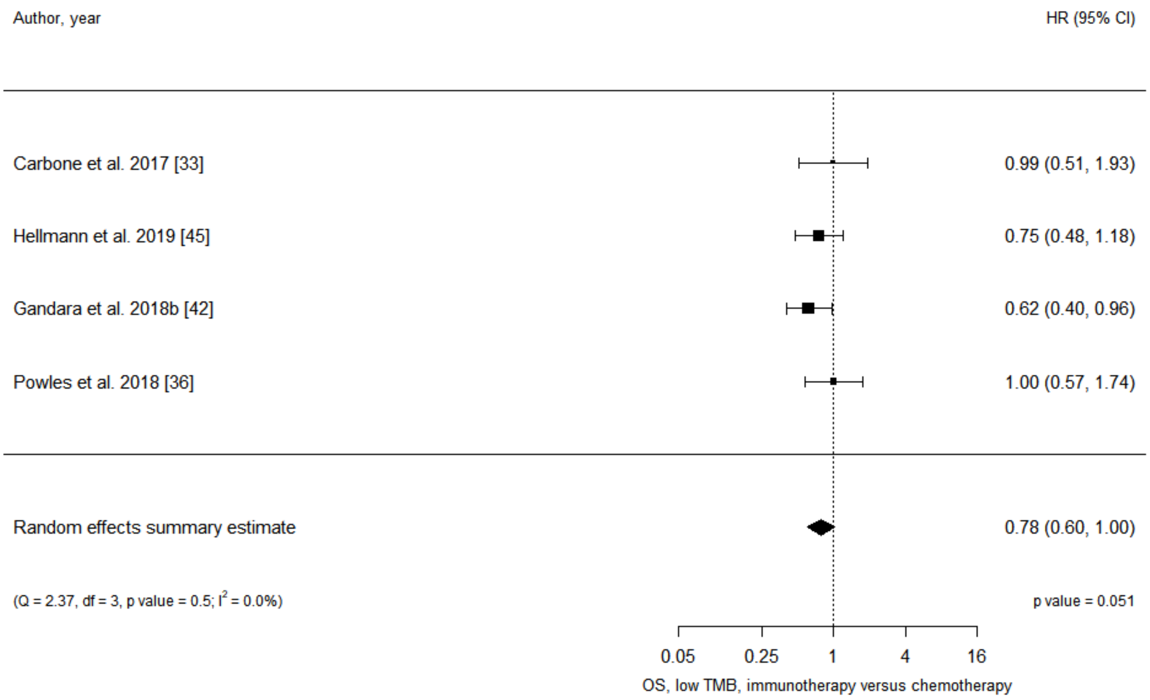

Figure S5. Overall survival, low TMB, immunotherapy vs chemotherapy, random-effects model.

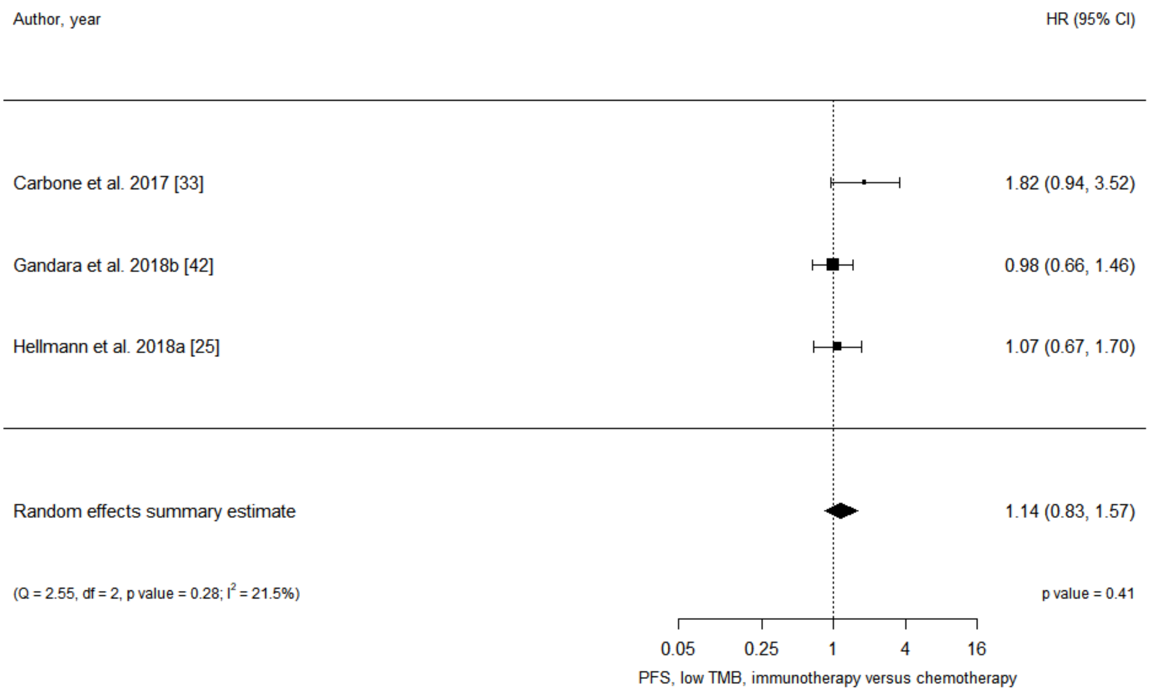

Figure S6. Progression-free survival, low TMB, immunotherapy vs chemotherapy, random-effects model.

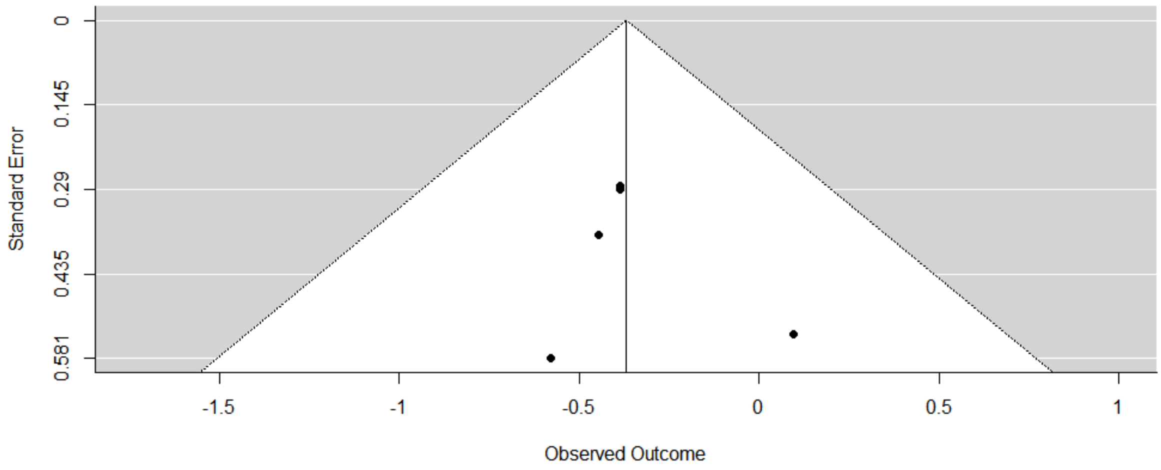

Figure S7. Overall survival, high TMB, immunotherapy vs chemotherapy, funnel plot.

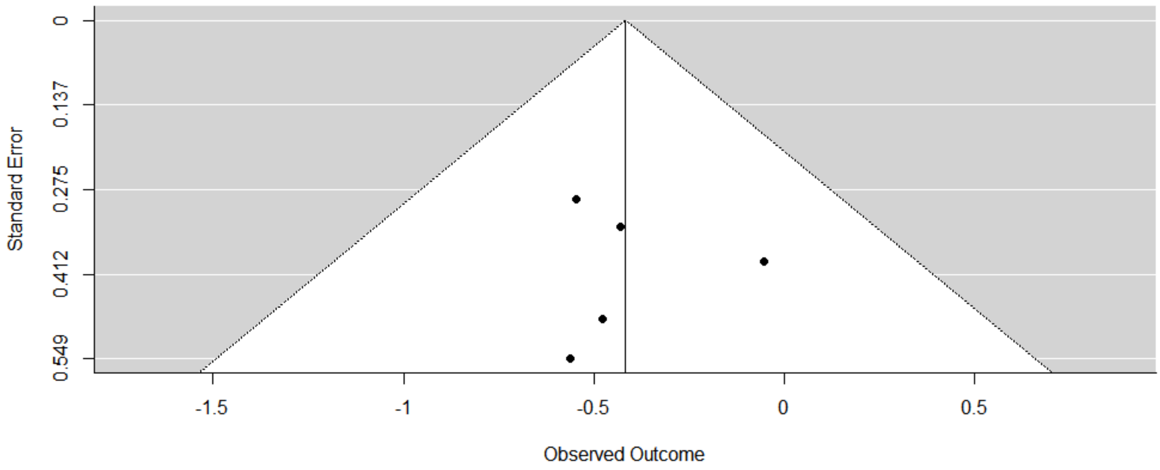

Figure S8. Progression-free survival, high TMB, immunotherapy vs chemotherapy, funnel plot.

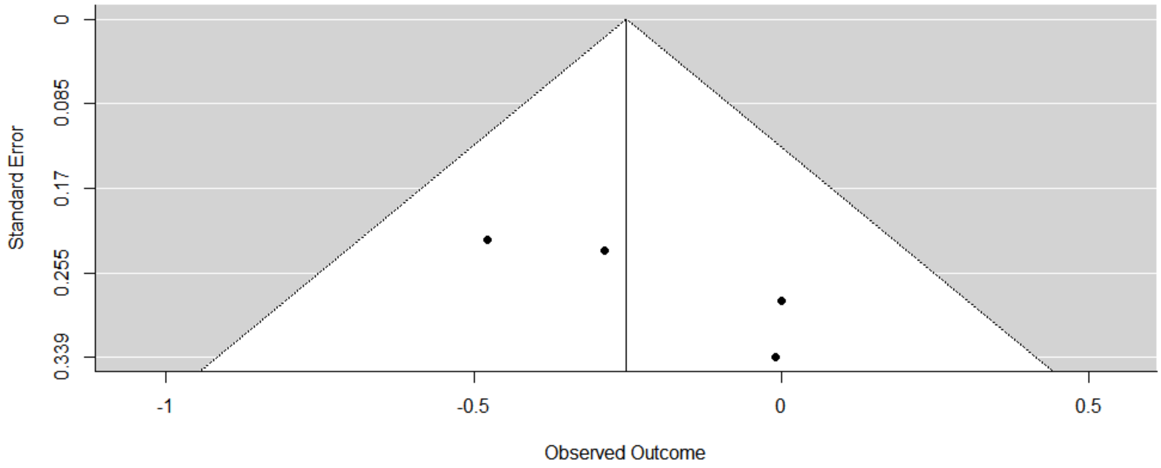

Figure S9. Overall survival, low TMB, immunotherapy vs chemotherapy, funnel plot.

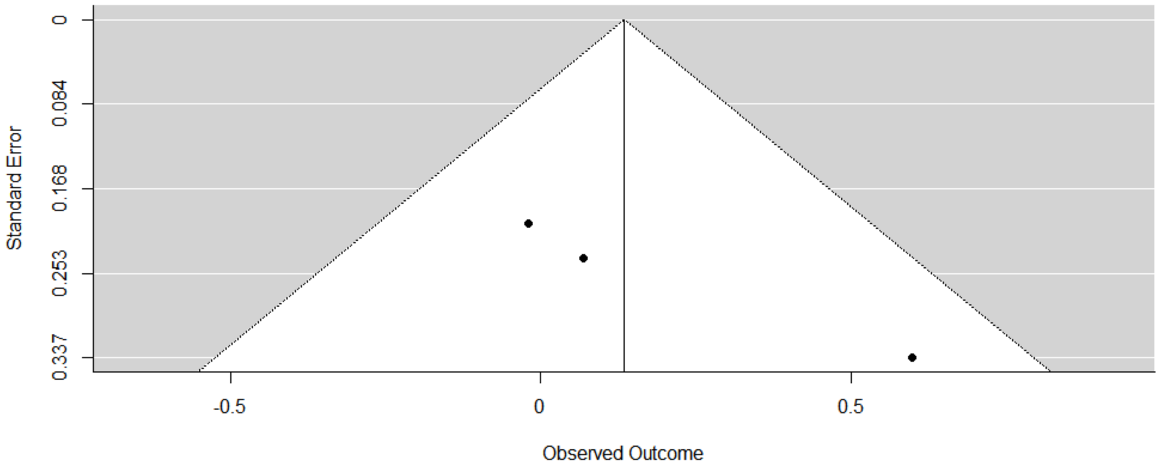

Figure S10. Progression-free survival, low TMB, immunotherapy vs chemotherapy, funnel plot.

© 2019 by the authors. Licensee MDPI, Basel, Switzerland. This article is an open access article distributed under the terms and conditions of the Creative Commons Attribution (CC BY) license (http://creativecommons.org/licenses/by/4.0/).
